# Supplementary material for: The Impact of Post-Mastectomy Radiotherapy on Survival Outcomes in Breast Cancer Patients Who Underwent Neoadjuvant Chemotherapy
Source: Cancers (Basel). 2021 Dec 9;13(24):6205. doi: 10.3390/cancers13246205 (PMC8699474; doi:10.3390/cancers13246205)
Supplement: Supplementary file 1 [file cancers-13-06205-s001.zip › cancers-1460747-supplementary.pdf]

cancers

MDPI

Supplementary Material: The Impact of Post-Mastectomy Radiotherapy on Survival Outcomes in Breast Cancer Patients Who Underwent Neoadjuvant Chemotherapy

Janghee Lee, Jee-Ye Kim, Soong-June Bae, Yeona Cho, Jung-Hwan Ji, Dooreh Kim, Sung-Gwe Ahn, Hyung-Seok Park, Seho Park, Seung-Il Kim, Byeong-Woo Park and Joon Jeong

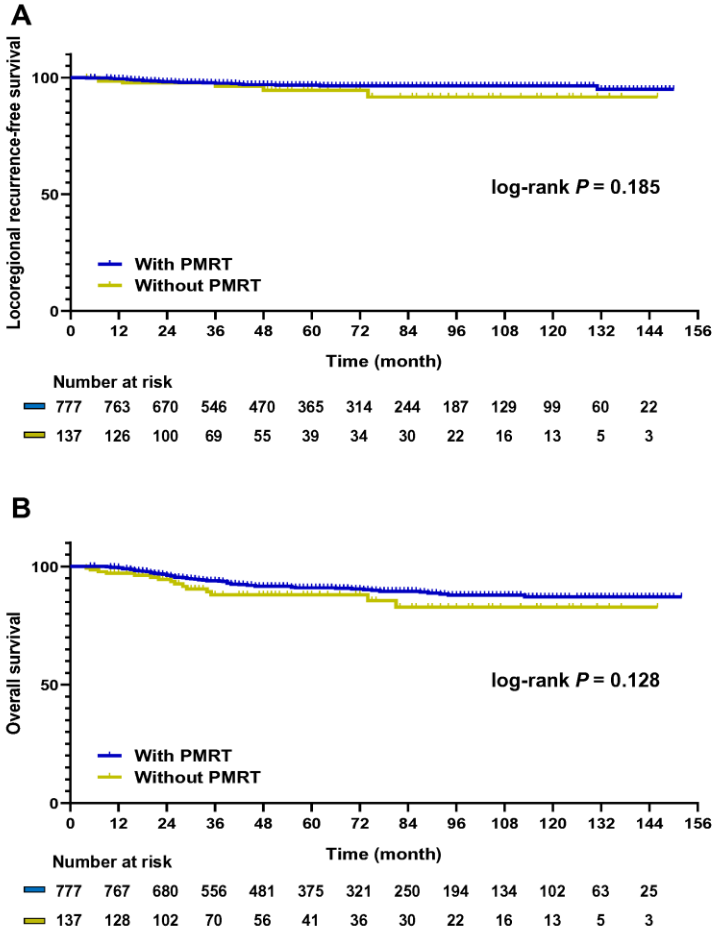

Figure S1. Kaplan–Meier survival curve of LRRFS and OS according to PMRT status in all patients. (**A**) LRRFS ($p = 0.185$); (**B**) OS ($p = 0.128$).

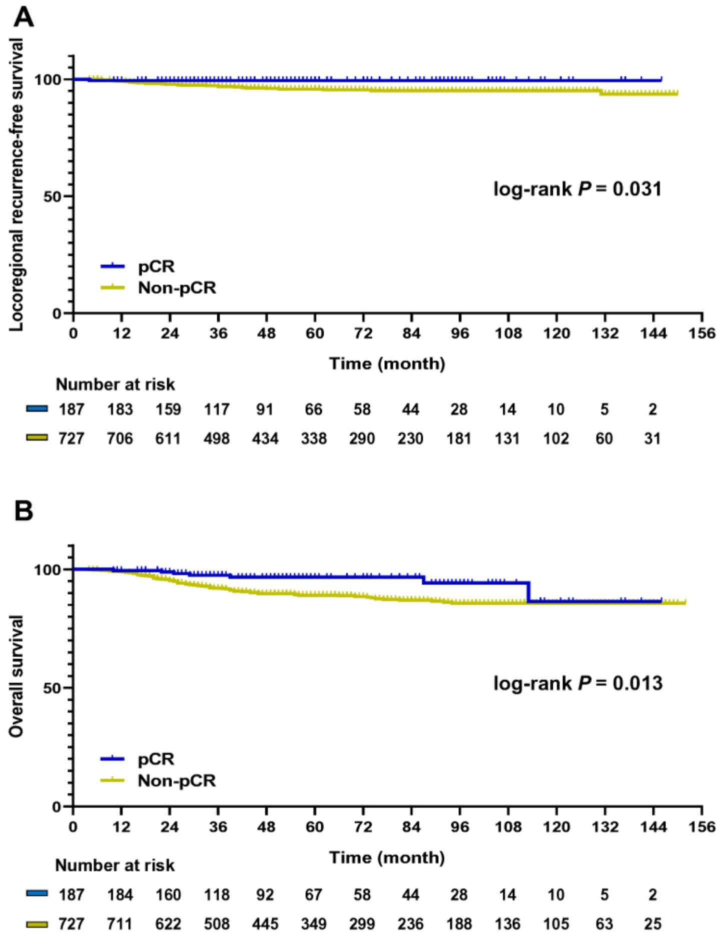

Figure S2. Kaplan–Meier survival curve of LRRFS and OS according to pCR status in all patients. (**A**) LRRFS (log-rank $p = 0.031$); (**B**) OS (log-rank $p = 0.013$).

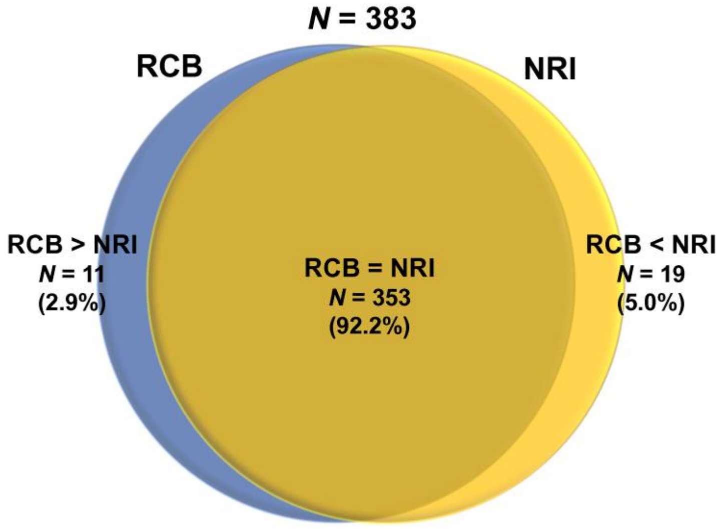

Figure S3. Comparison between RCB index and NRI.

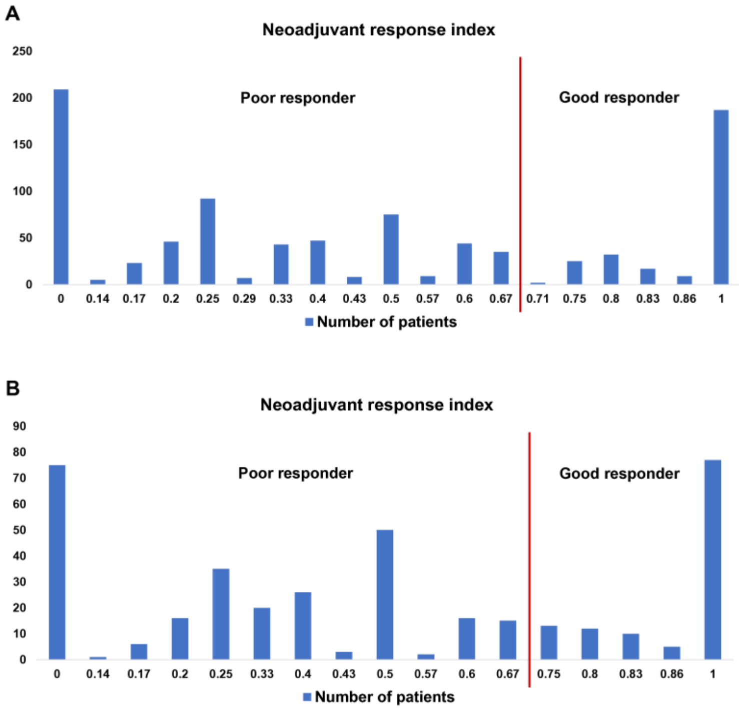

Figure S4. Distribution of patients' NRI. Scores range from 0 to 1, with 1 indicating pCR and 0 indicating SD or PD. A good responder was defined as having 0.7 < NRI ≤ 1, while a poor responder was defined as having NRI < 0.7. (**A**) All patients; (**B**) 1:3 PSM patients.

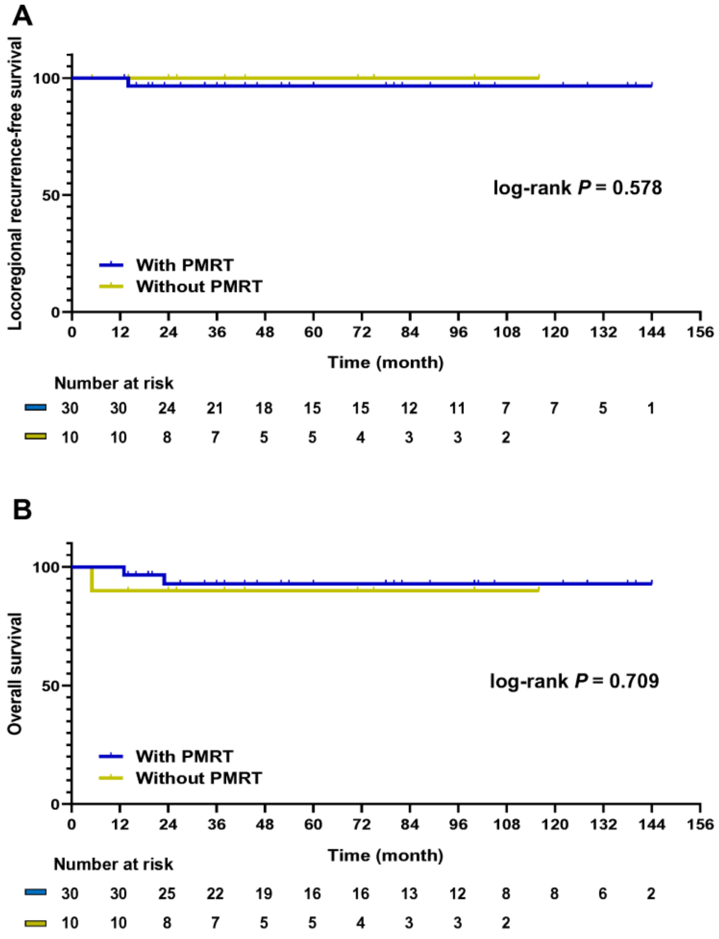

Figure S5. Kaplan–Meier survival curve of LRRFS and OS according to PMRT status in the good responder group, except those of the 1:3 PSM patients who achieved pCR. (**A**) LRRFS (p = 0.578); (**B**) OS (p = 0.709).

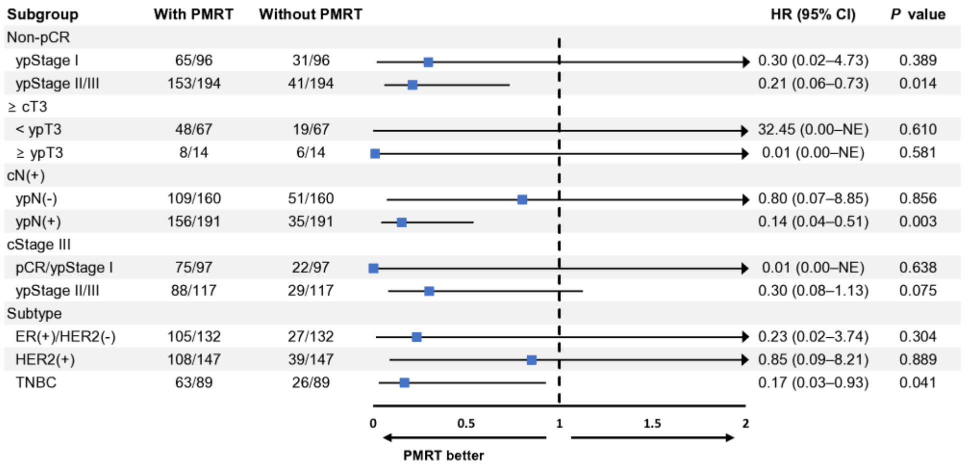

Figure S6. Subgroup analysis of LRRFS according to PMRT status in 1:3 PSM patients.

Table S1. Calculation algorithm of NRI.

NRI calculation	
$NRI^1 = \frac{(A-B+C)+(D-E)}{(A+1)+D} = \frac{\text{Breast response score + Nodal response score}}{\text{Sum of maximal achievable response score}}$	
	Breast response score
A	cT stage
B	ypT stage[2]
C	Response based on pathology
	0 = No pCR<br>1 = Near pCR (tumor size < 5mm)<br>2 = pCR
	Nodal response score
D	cN stage
E	ypN stage[3]

[1]In case of progressive disease NRI = 0; [2]Except, ypT0 or ypTis =1, not 0; [3]Only isolated tumor cells (LN ≤ 0.2mm) was classified as ypN0, and micrometastases (0.2mm < LN ≤ 2mm) was considered as ypN1; NRI, neoadjuvant response index; pCR, pathologic complete response; LN, lymph node.

Table S2. pCR rate in patients with or without PMRT according to breast cancer subtype.

Variable	All patients (%)	Patients with PMRT (%)	Patients without PMRT (%)	*P* value
ER-positive/HER2-negative	-	-	-	0.419
pCR	25 (6.5)	1 (3.1)	24 (6.8)	-
Non-pCR	360 (93.5)	31 (96.9)	329 (93.2)	-
HER2-positive	-	-	-	<0.001
pCR	117 (34.6)	36 (53.7)	81 (29.9)	-
Non-pCR	221 (65.4)	31 (46.3)	190 (70.1)	-
TNBC	-	-	-	0.084
pCR	45 (23.6)	13 (34.2)	32 (20.9)	-
Non-pCR	146 (76.4)	25 (65.8)	121 (79.1)	-

pCR, pathologic complete response; PMRT, post-mastectomy radiotherapy; ER, estrogen receptor; HER2, human epidermal growth factor receptor 2; TNBC, triple negative breast cancer.

Table S3. Multivariate analysis of LRRFS and OS in all patients with or without pCR after NAC.

Variable	LRRFS						OS					
	All patients		pCR group		non-pCR group		All patients		pCR group		non-pCR group	
	HR (95% CI)	*P* value	HR (95% CI)	*P* value	HR (95% CI)	*P* value	HR (95% CI)	*P* value	HR (95% CI)	*P* value	HR (95% CI)	*P* value
Age at diagnosis	1.00 (0.97–1.04)	0.891	1.34 (0.48–3.75)	0.582	1.00 (0.97–1.04)	0.859	1.02 (1.00–1.05)	0.039	1.07 (0.99–1.15)	0.105	1.02 (1.00–1.04)	0.098
cT Stage												
cT1	Ref.*		Ref.		Ref.		Ref.		Ref.		Ref.	
cT2	4.14 (0.55–31.28)	0.169	NE		NE		0.77 (0.41–1.45)	0.414	1.15 (0.19–7.07)	0.879	0.78 (0.39–1.54)	0.475
cT3	4.59 (0.58–36.36)	0.149	NE		NE		1.05 (0.54–2.06)	0.89	0.82 (0.06–10.50)	0.880	1.11 (0.54–2.28)	0.778
cN stage												
cN0	Ref.		Ref.		Ref.		Ref,		Ref.		Ref.	
cN+	NE		NE		NE		NE		NE		NE	
ER												
Positive	Ref.		Ref.		Ref.		Ref.		Ref.		Ref.	
Negative	5.68 (1.88–17.15)	0.002	NE		5.62 (1.84–17.11)	0.002	3.37 (1.81–6.25)	<0.001	1.27 (0.14–11.40)	0.831	3.48(1.84–6.60)	<0.001
PR												
Positive	Ref.		Ref.		Ref.		Ref.		Ref.		Ref.	
Negative	0.55 (0.18-*1.65)	0.287	NE		0.54 (0.18–1.63)	0.272	1.65 (0.83–3.29)	0.155	NE		1.52 (0.76–3.07)	0.239
HER2												
Negative	Ref.		Ref.		Ref.		Ref.		Ref.		Ref.	
Positive	0.90 (0.40–2.05)	0.808	NE		0.88 (0.38–2.05)	0.769	0.77 (0.48–1.24)	0.285	0.21 (0.04–1.24)	0.085	0.89 (0.54–1.47)	0.655
ypStage												
pCR	Ref.						Ref.					
Stage I	3.98 (0.44–36.04)	0.219			Ref.		1.23 (0.44–3.42)	0.692			Ref.	
Stage II	8.85 (1.08–72.69)	0.043			2.26 (0.68–7.54)	0.185	3.39 (1.42–8.07)	0.006			2.84 (1.26–6.40)	0.012
Stage III	26.69 (3.28–217.43)	0.002			7.06 (2.17–22.99)	0.001	11.76 (5.07–27.32)	0.001			10.00 (4.61–21.69)	<0.001
PMRT												
No	Ref.		Ref.		Ref.		Ref.		Ref.		Ref.	
Yes	0.27 (0.11–0.71)	0.008	NE		0.24 (0.09–0.62)	0.003	0.38 (0.21–0.68)	0.001	0.47 (0.08–2.79)	0.409	0.34 (0.18–0.63)	0.001

*Reference value; LRRFS, locoregional recurrence-free survival; OS, overall survival; pCR, pathologic complete response; NAC, neoadjuvant chemotherapy; HR, hazard ratio; CI, confidence intervals, NE, not estimated; PMRT, ER, estrogen receptor; PR, progesterone receptor; HER2, human epidermal growth factor receptor 2; PMRT, post-mastectomy radiotherapy.

Table S4. Multivariate analysis of LRRFS and OS in all patients according to NRI.

Variable	LRRFS				OS			
	NRI < 0.7		NRI > 0.7		NRI < 0.7		NRI > 0.7	
	HR (95% CI)	*P* value	HR (95% CI)	*P* value	HR (95% CI)	*P* value	HR (95% CI)	*P* value
Age at diagnosis	1.01 (0.97–1.05)	0.768	1.05 (0.80–1.38)	0.741	1.02 (0.99–1.04)	0.166	2.17 (0.75–6.31)	0.153
cT Stage								
cT1	Ref.*		Ref.		Ref.		Ref.	
cT2	NE		NE		0.91 (0.45–1.85)	0.788	NE	
cT3	NE		NE		1.20 (0.57–2.55)	0.633	NE	
cN Stage								
cN0	Ref.		Ref.		Ref.		Ref.	
cN+	NE		NE		NE		NE	
ER								
Positive	Ref.		Ref.		Ref.		Ref.	
Negative	5.26 (1.73–15.95)	0.003	NE		3.31 (1.74–6.31)	<0.001	NE	
PR								
Positive	Ref.		Ref.		Ref.		Ref.	
Negative	0.54 (0.18–1.63)	0.273	NE		1.52 (0.75–3.07)	0.246	NE	
HER2								
Negative	Ref.		Ref.		Ref.		Ref.	
Positive	0.91 (0.38–2.18)	0.830	NE		0.99 (0.59–1.64)	0.953	2.71 (0.01–1545.35)	0.758
ypStage								
pCR			Ref.				Ref.	
Stage I	Ref.		NE		Ref.		NE	
Stage II	1.37 (0.40–4.64)	0.616			2.39 (0.96–5.95)	0.062		
Stage III	4.56 (1.42–14.69)	0.011			8.70 (3.66–20.68)	<0.001		
PMRT								
No	Ref.		Ref.		Ref.		Ref.	
Yes	0.21 (0.08–0.57)	0.002	NE	NE	0.33 (0.17–0.62)	0.001	NE	

*Reference value, LRRFS, locoregional recurrence-free survival; OS, overall survival; NRI, neoadjuvant response index; HR, hazard ratio; CI, confidence intervals, NE, not estimated; pCR, pathologic complete response; ER, estrogen receptor; PR, progesterone receptor; HER2, human epidermal growth factor receptor 2; PMRT, post-mastectomy radiotherapy.
